# Supplementary material for: Assessing the Differential Methylation Analysis Quality for Microarray and NGS Platforms
Source: Int J Mol Sci. 2023 May 11;24(10):8591. doi: 10.3390/ijms24108591 (PMC10218268; doi:10.3390/ijms24108591)
Supplement: Supplementary file 1 [file ijms-24-08591-s001.zip › ijms-2368233-supplementary.pdf]

Supplementary

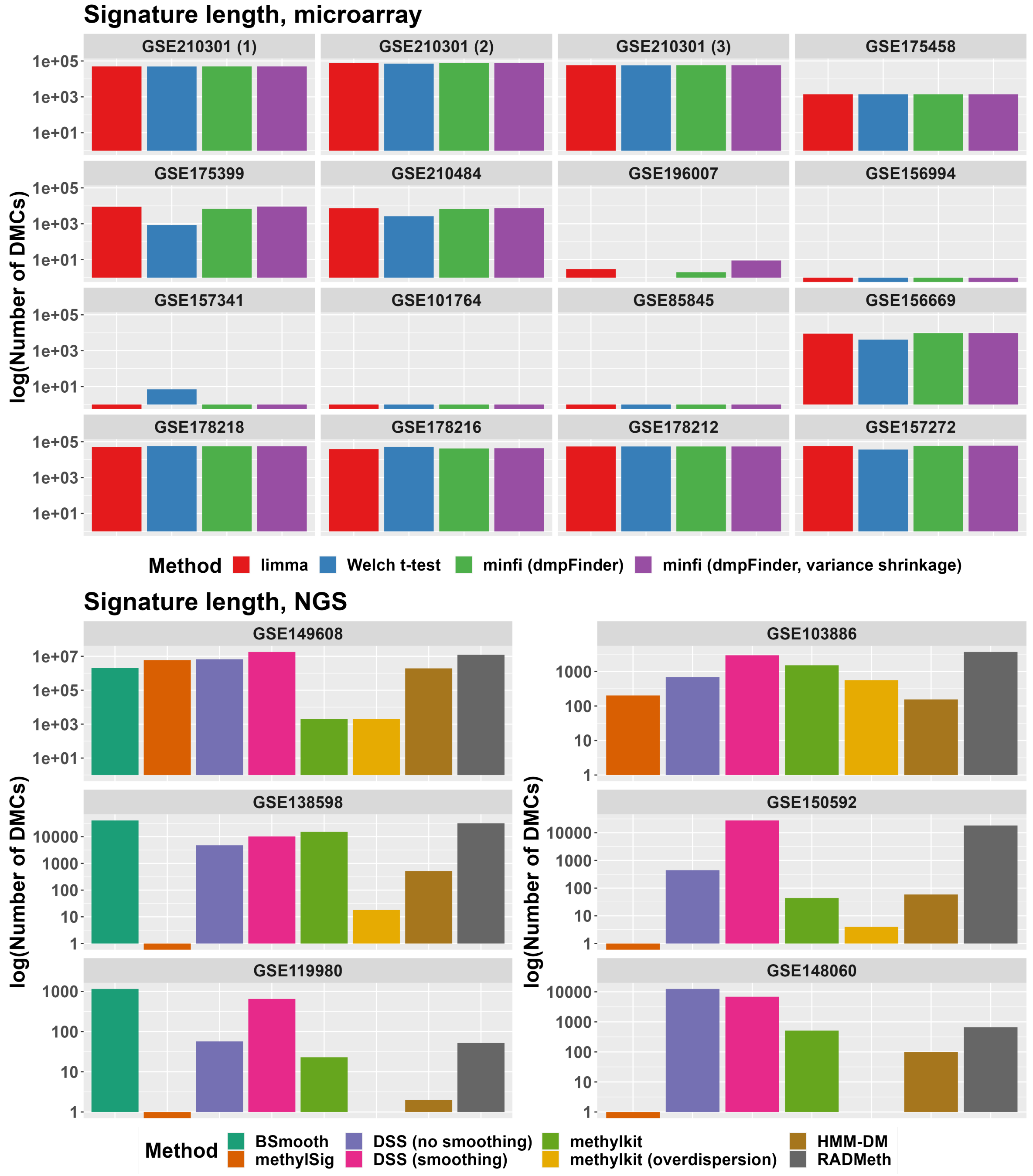

Supplementary Figure S1: The number of DMPs or DMCs for each evaluated method and dataset (log scale).

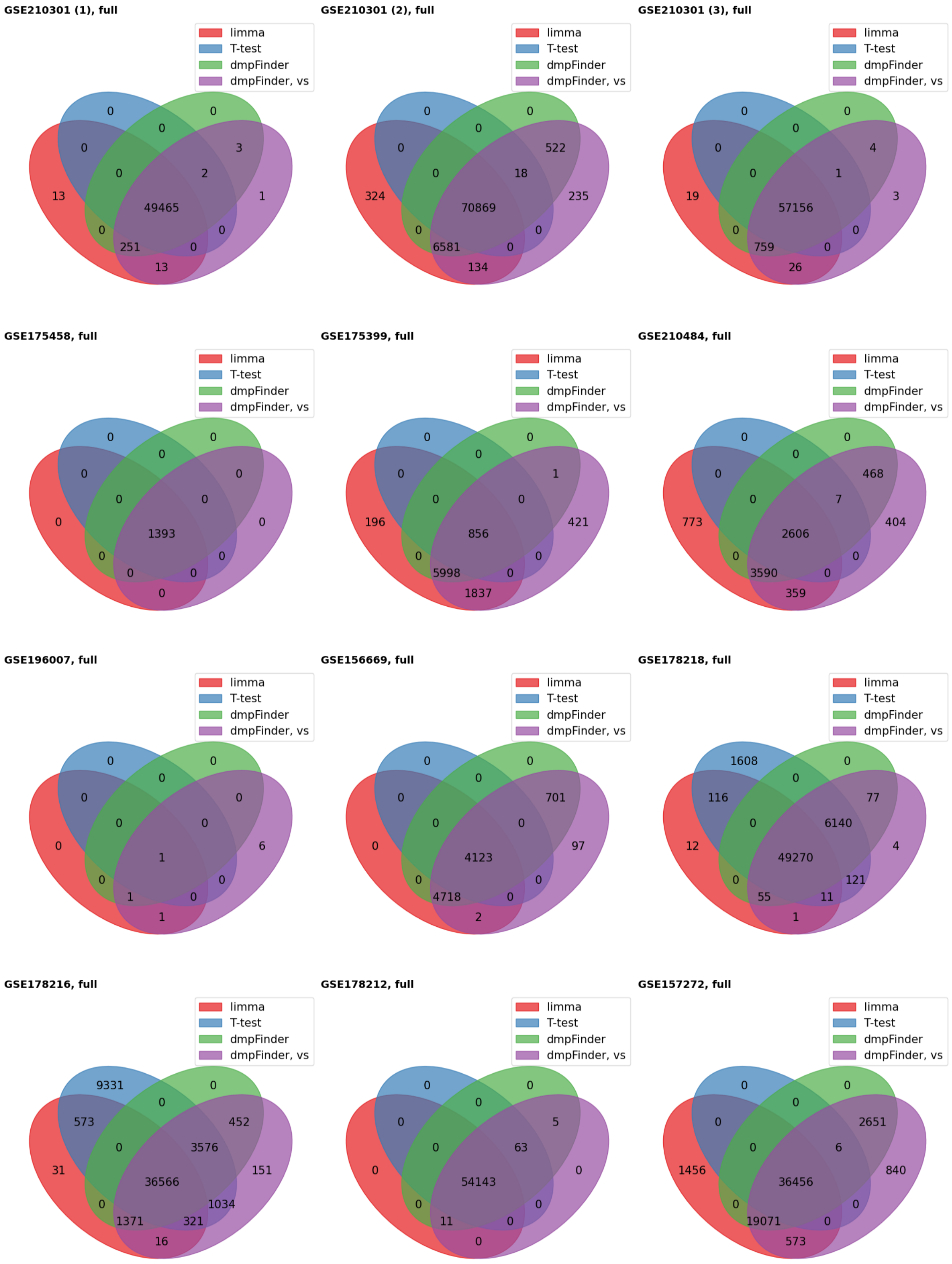

Supplementary Figure S2: Venn diagrams for the full signatures of microarray DM methods.

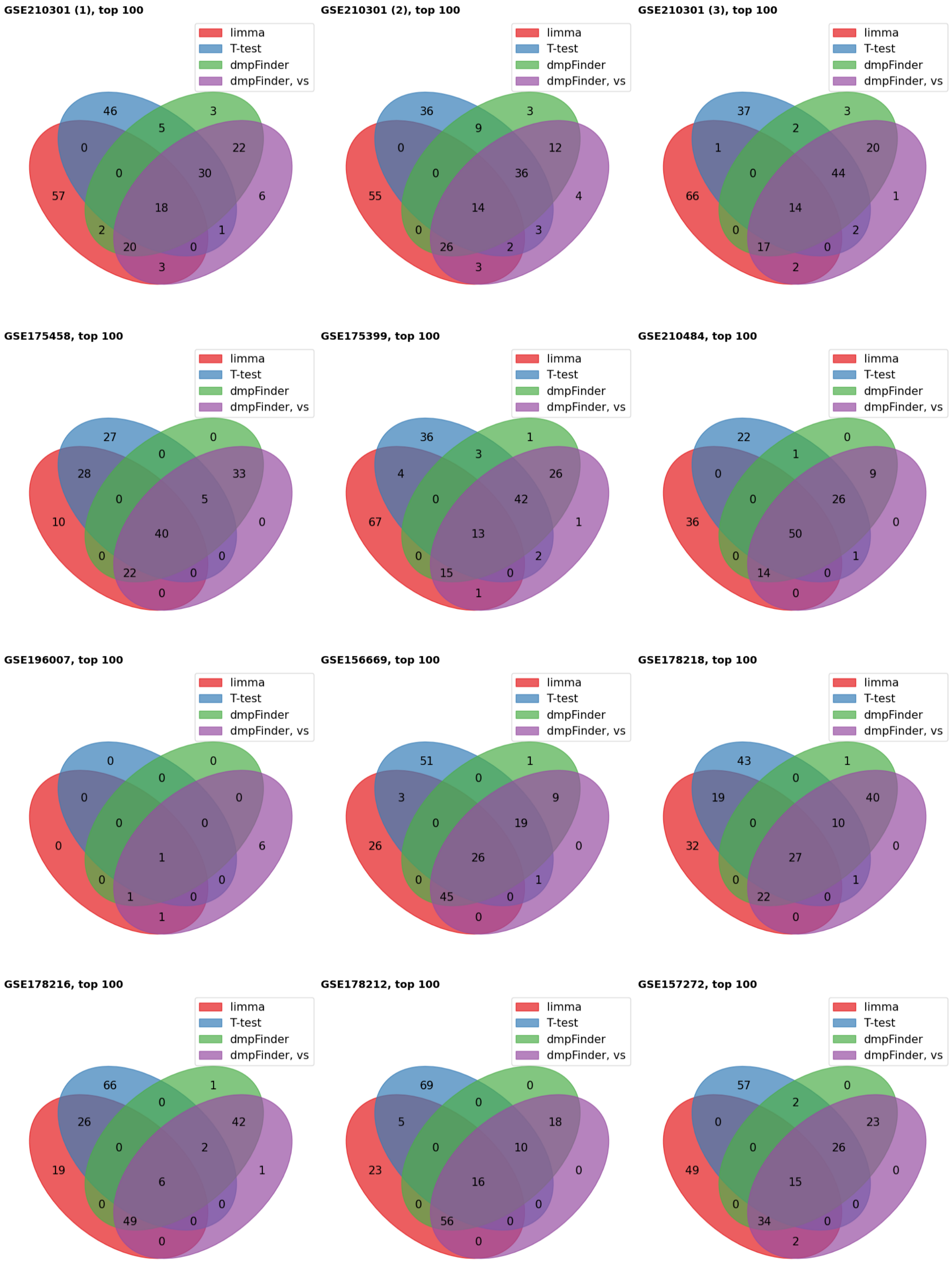

Supplementary Figure S3: Venn diagrams for the top 100 DMP signatures of microarray DM methods.

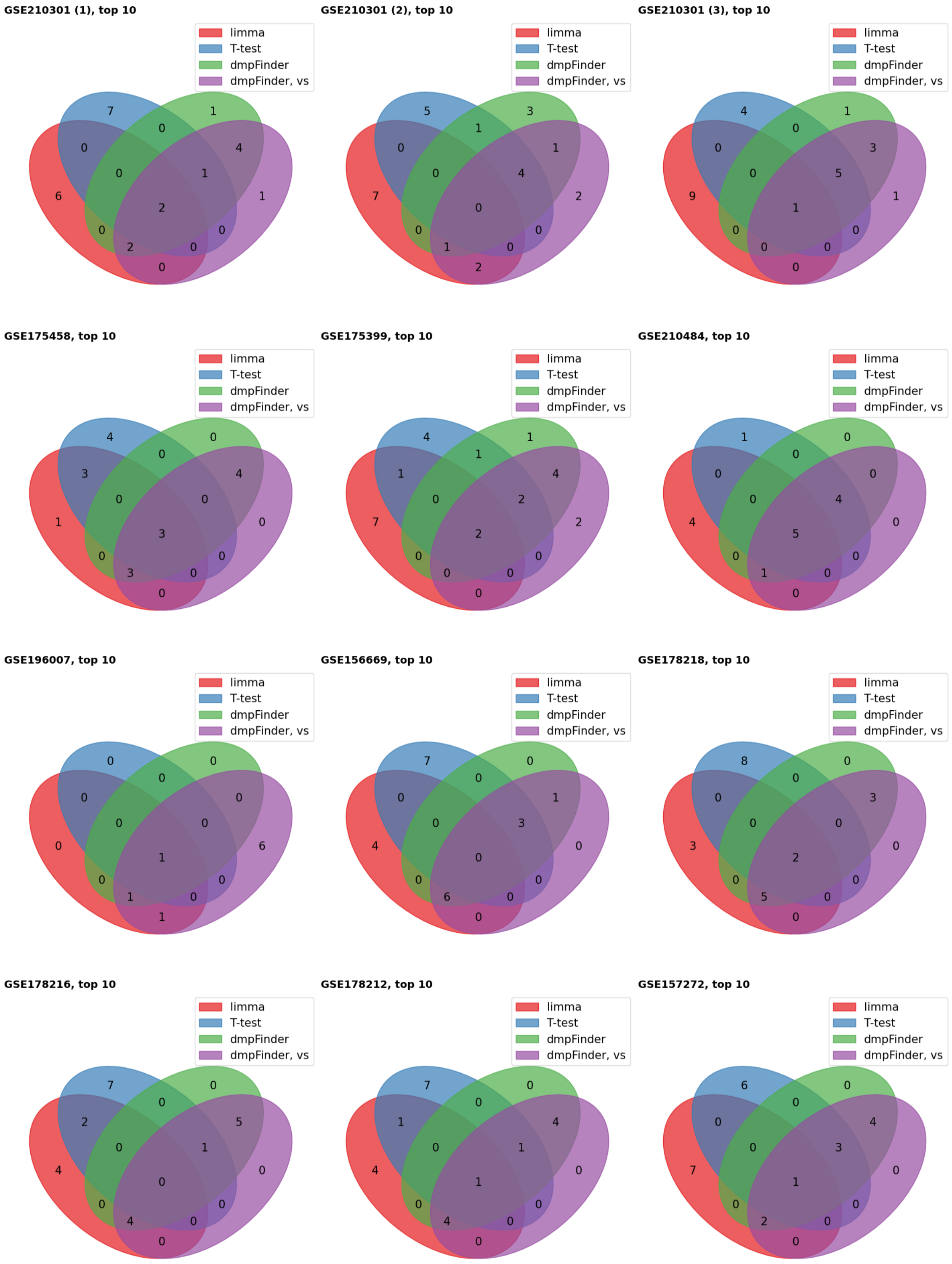

Supplementary Figure S4: Venn diagrams for the top 10 DMP signatures of microarray DM methods.

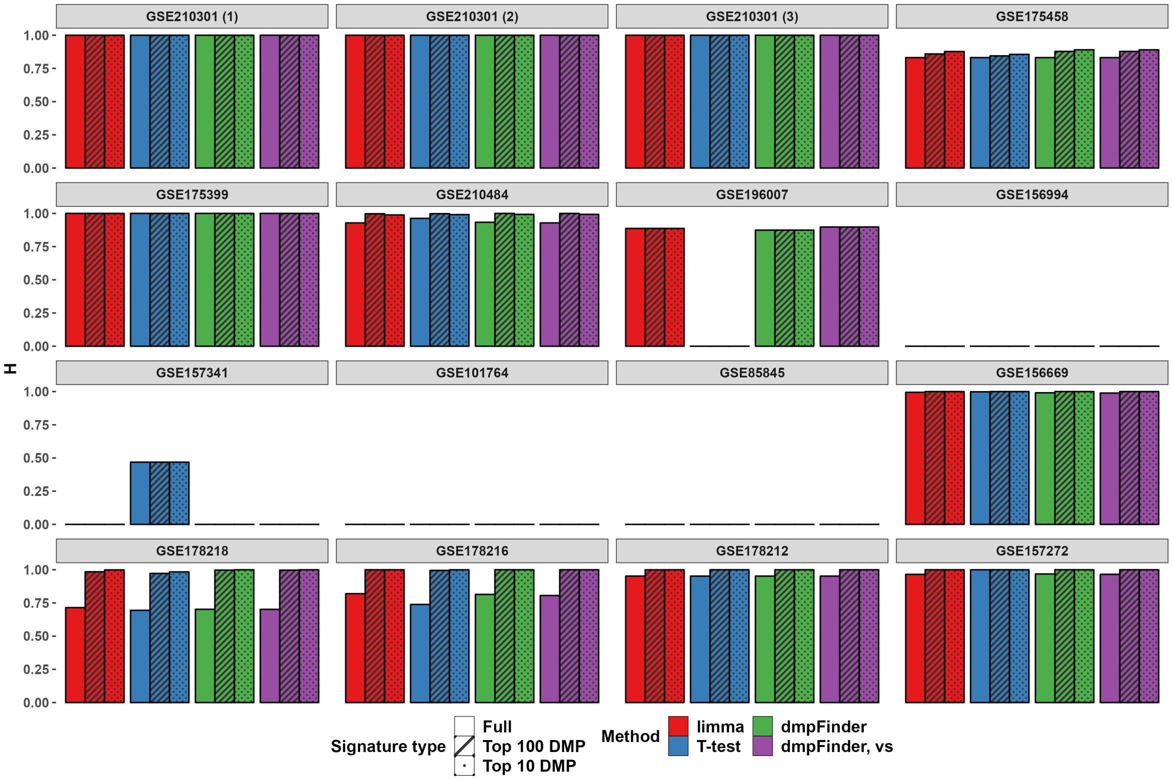

Supplementary Figure S5: Hobotnica metric values for each microarray dataset.

Supplementary Table S1: H-scores, lengths, and P-values of the full microarray signatures

Dataset	Method	Length	H-score	P-value
GSE210301 (1)	limma	49742	1	1
	T-test	49467	1	1
	dmpFinder	49721	1	1
	dmpFinder (variance shrinkage)	49735	1	1
GSE210301 (2)	limma	77908	1	2e-04
	T-test	70887	1	2e-04
	dmpFinder	77990	1	2e-04
	dmpFinder (variance shrinkage)	78359	1	2e-04
GSE210301 (3)	limma	57960	1	1
	T-test	57157	1	1
	dmpFinder	57920	1	1
	dmpFinder (variance shrinkage)	57949	1	1
GSE175458	limma	1393	0.8314	2e-04
	T-test	1393	0.8314	2e-04
	dmpFinder	1393	0.8314	2e-04
	dmpFinder (variance shrinkage)	1393	0.8314	2e-04
GSE156994	limma	0	0	NA
	T-test	0	0	NA
	dmpFinder	0	0	NA
	dmpFinder (variance shrinkage)	0	0	NA
GSE157341	limma	0	0	NA
	T-test	7	0.4684	0.953
	dmpFinder	0	0	NA
	dmpFinder (variance shrinkage)	0	0	NA
GSE85845	limma	0	0	NA
	T-test	0	0	NA
	dmpFinder	0	0	NA
	dmpFinder (variance shrinkage)	0	0	NA
GSE175399	limma	8887	1	0.0292
	T-test	856	1	0.0322
	dmpFinder	6855	1	0.0316
	dmpFinder (variance shrinkage)	9113	1	0.0262
GSE210484	limma	7328	0.9286	2e-04
	T-test	2613	0.9626	2e-04
	dmpFinder	6671	0.9331	2e-04
	dmpFinder (variance shrinkage)	7434	0.9286	2e-04
GSE196007	limma	3	0.8869	2e-04
	T-test	1	0	NA
	dmpFinder	2	0.8742	2e-04
	dmpFinder (variance shrinkage)	9	0.8976	2e-04
GSE156669	limma	8843	0.9945	0.001
	T-test	4123	0.9982	0.0012
	dmpFinder	9542	0.9908	0.0014
	dmpFinder (variance shrinkage)	9641	0.9889	0.0014
GSE178218	limma	49465	0.7148	0.0226
	T-test	57266	0.6944	0.0374
	dmpFinder	55542	0.7022	0.0344
	dmpFinder (variance shrinkage)	55679	0.7018	0.0328
GSE178216	limma	38878	0.8197	0.0084
	T-test	51401	0.7387	0.0442
	dmpFinder	41965	0.8144	0.0106
	dmpFinder (variance shrinkage)	43487	0.806	0.0106
GSE178212	limma	54154	0.9524	0.0042
	T-test	54206	0.9522	0.003
	dmpFinder	54222	0.9522	0.0036
	dmpFinder (variance shrinkage)	54222	0.9522	0.0038
GSE157272	limma	57556	0.9652	0.0318
	T-test	36462	0.9997	0.0034
	dmpFinder	58184	0.9682	0.0264
	dmpFinder (variance shrinkage)	59597	0.9656	0.0348

Supplementary Table S2: H-scores, lengths, and P-values of the top 100 DMP microarray signatures.

Dataset	Method	Length	H-score	P-value
GSE210301 (1)	limma	100	1	1
	T-test	100	1	1
	dmpFinder	100	1	1
	dmpFinder (variance shrinkage)	100	1	1
GSE210301 (2)	limma	100	1	2e-04
	T-test	100	1	2e-04
	dmpFinder	100	1	2e-04
	dmpFinder (variance shrinkage)	100	1	2e-04
GSE210301 (3)	limma	100	1	1
	T-test	100	1	1
	dmpFinder	100	1	1
	dmpFinder (variance shrinkage)	100	1	1
GSE175458	limma	100	0.8589	2e-04
	T-test	100	0.8444	2e-04
	dmpFinder	100	0.8781	2e-04
	dmpFinder (variance shrinkage)	100	0.8781	NA
GSE156994	limma	0	0	NA
	T-test	0	0	NA
	dmpFinder	0	0	NA
	dmpFinder (variance shrinkage)	0	0	NA
GSE157341	limma	0	0	NA
	T-test	7	0.4684	0.953
	dmpFinder	0	0	NA
	dmpFinder (variance shrinkage)	0	0	NA
GSE85845	limma	0	0	NA
	T-test	0	0	NA
	dmpFinder	0	0	NA
	dmpFinder (variance shrinkage)	0	0	NA
GSE175399	limma	100	1	0.0308
	T-test	100	1	0.0316
	dmpFinder	100	1	0.0292
	dmpFinder (variance shrinkage)	100	1	0.0344
GSE210484	limma	100	0.9973	2e-04
	T-test	100	0.9978	2e-04
	dmpFinder	100	0.9998	2e-04
	dmpFinder (variance shrinkage)	100	0.9998	2e-04
GSE196007	limma	3	0.8869	2e-04
	T-test	1	0	NA
	dmpFinder	2	0.8742	2e-04
	dmpFinder (variance shrinkage)	9	0.8976	2e-04
GSE156669	limma	100	1	0.0012
	T-test	100	1	8e-04
	dmpFinder	100	1	2e-04
	dmpFinder (variance shrinkage)	100	1	2e-04
GSE178218	limma	100	0.9846	2e-04
	T-test	100	0.9722	2e-04
	dmpFinder	100	0.9972	2e-04
	dmpFinder (variance shrinkage)	100	0.997	2e-04
GSE178216	limma	100	0.9999	2e-04
	T-test	100	0.9949	2e-04
	dmpFinder	100	1	2e-04
	dmpFinder (variance shrinkage)	100	1	2e-04
GSE178212	limma	100	0.9996	2e-04
	T-test	100	1	2e-04
	dmpFinder	100	0.9997	2e-04
	dmpFinder (variance shrinkage)	100	0.9997	2e-04
GSE157272	limma	100	1	0.0018
	T-test	100	1	0.003
	dmpFinder	100	1	0.0022
	dmpFinder (variance shrinkage)	100	1	0.0018

Supplementary Table S3: H-scores, lengths, and P-values of the top 10 DMP microarray signatures.

Dataset	Method	Length	H-score	P-value
GSE210301 (1)	limma	10	1	1
	T-test	10	1	1
	dmpFinder	10	1	1
	dmpFinder (variance shrinkage)	10	1	1
GSE210301 (2)	limma	10	1	2e-04
	T-test	10	1	2e-04
	dmpFinder	10	1	2e-04
	dmpFinder (variance shrinkage)	10	1	2e-04
GSE210301 (3)	limma	10	1	1
	T-test	10	1	1
	dmpFinder	10	1	1
	dmpFinder (variance shrinkage)	10	1	1
GSE175458	limma	10	0.8773	2e-04
	T-test	10	0.8557	2e-04
	dmpFinder	10	0.8903	2e-04
	dmpFinder (variance shrinkage)	10	0.8903	NA
GSE156994	limma	0	0	NA
	T-test	0	0	NA
	dmpFinder	0	0	NA
	dmpFinder (variance shrinkage)	0	0	NA
GSE157341	limma	0	0	NA
	T-test	7	0.4684	0.953
	dmpFinder	0	0	NA
	dmpFinder (variance shrinkage)	0	0	NA
GSE85845	limma	0	0	NA
	T-test	0	0	NA
	dmpFinder	0	0	NA
	dmpFinder (variance shrinkage)	0	0	NA
GSE175399	limma	10	1	0.0314
	T-test	10	1	0.034
	dmpFinder	10	1	0.0308
	dmpFinder (variance shrinkage)	10	1	0.0296
GSE210484	limma	10	0.9885	2e-04
	T-test	10	0.991	2e-04
	dmpFinder	10	0.9927	2e-04
	dmpFinder (variance shrinkage)	10	0.9927	2e-04
GSE196007	limma	3	0.8869	2e-04
	T-test	1	0	NA
	dmpFinder	2	0.8742	2e-04
	dmpFinder (variance shrinkage)	9	0.8976	2e-04
GSE156669	limma	10	1	2e-04
	T-test	10	1	6e-04
	dmpFinder	10	1	4e-04
	dmpFinder (variance shrinkage)	10	1	4e-04
GSE178218	limma	10	0.9991	2e-04
	T-test	10	0.9839	2e-04
	dmpFinder	10	0.9999	2e-04
	dmpFinder (variance shrinkage)	10	0.9999	2e-04
GSE178216	limma	10	1	2e-04
	T-test	10	0.9998	2e-04
	dmpFinder	10	1	2e-04
	dmpFinder (variance shrinkage)	10	1	2e-04
GSE178212	limma	10	0.9999	2e-04
	T-test	10	1	2e-04
	dmpFinder	10	1	2e-04
	dmpFinder (variance shrinkage)	10	1	2e-04
GSE157272	limma	10	1	0.0022
	T-test	10	1	0.003
	dmpFinder	10	1	0.0038
	dmpFinder (variance shrinkage)	10	1	0.0018

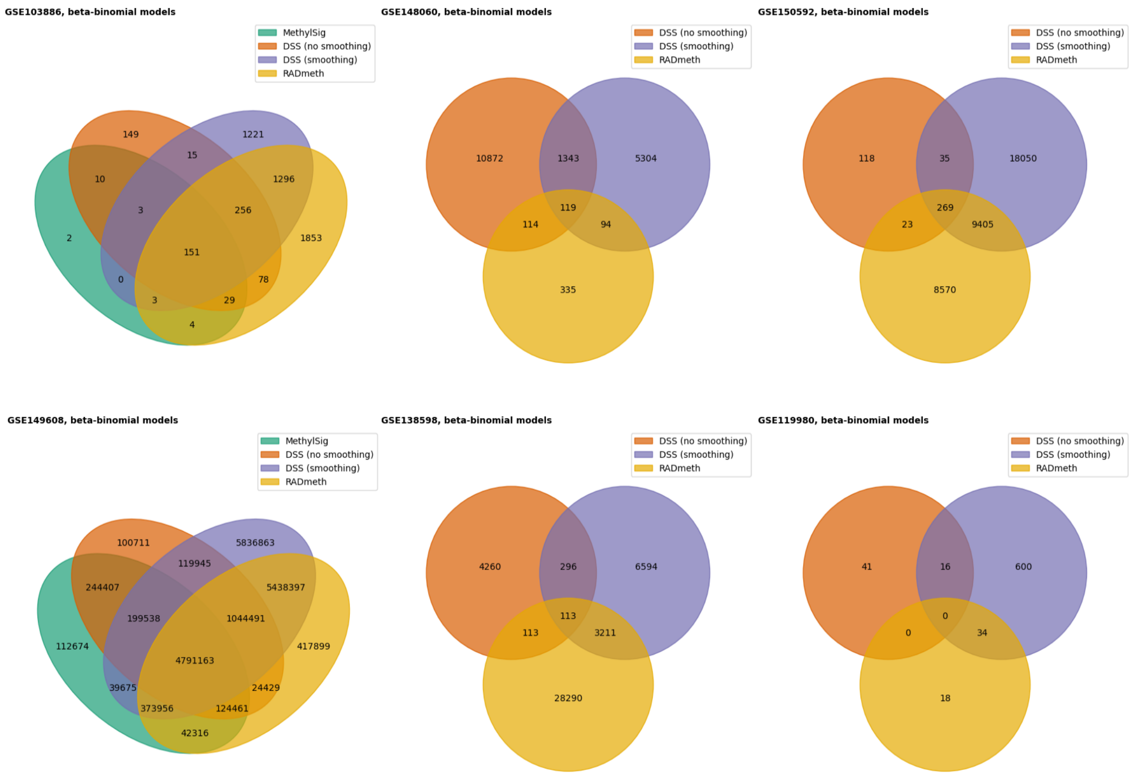

Supplementary Figure S6: Venn diagrams for NGS beta-binomial DM methods (the full signatures).

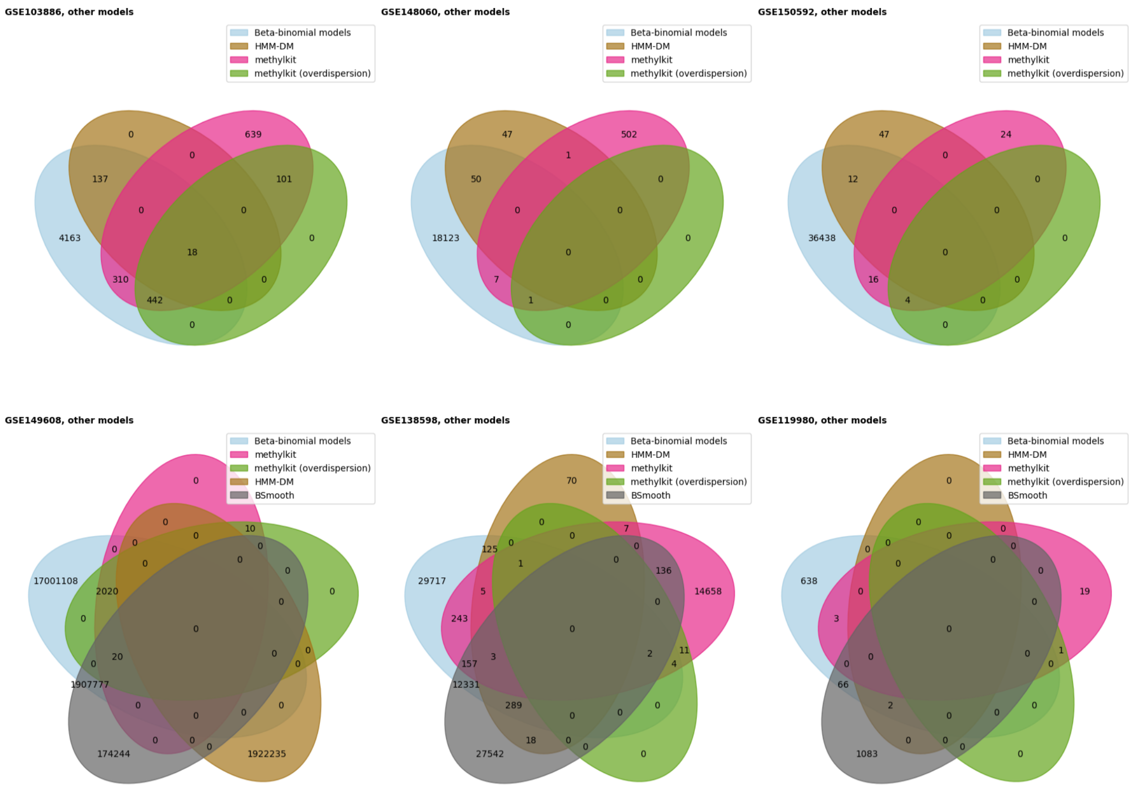

Supplementary Figure S7: Venn diagrams for the union of beta-binomial methods DMCs (blue) and other evaluated NGS methods (the full signatures). The intersection of all methods is empty for all datasets except GSE103886 and 12 DMCs for GSE103886.

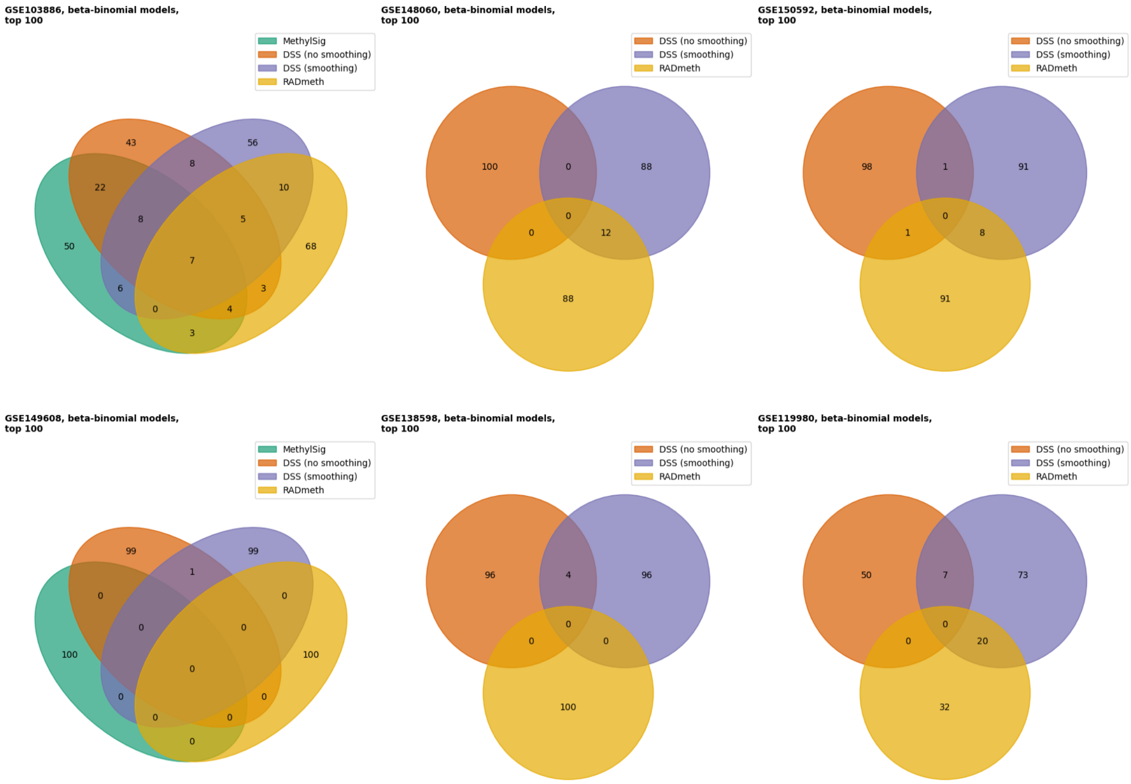

Supplementary Figure S8: Venn diagrams for NGS beta-binomial DM methods (the top 100 DMC signatures).

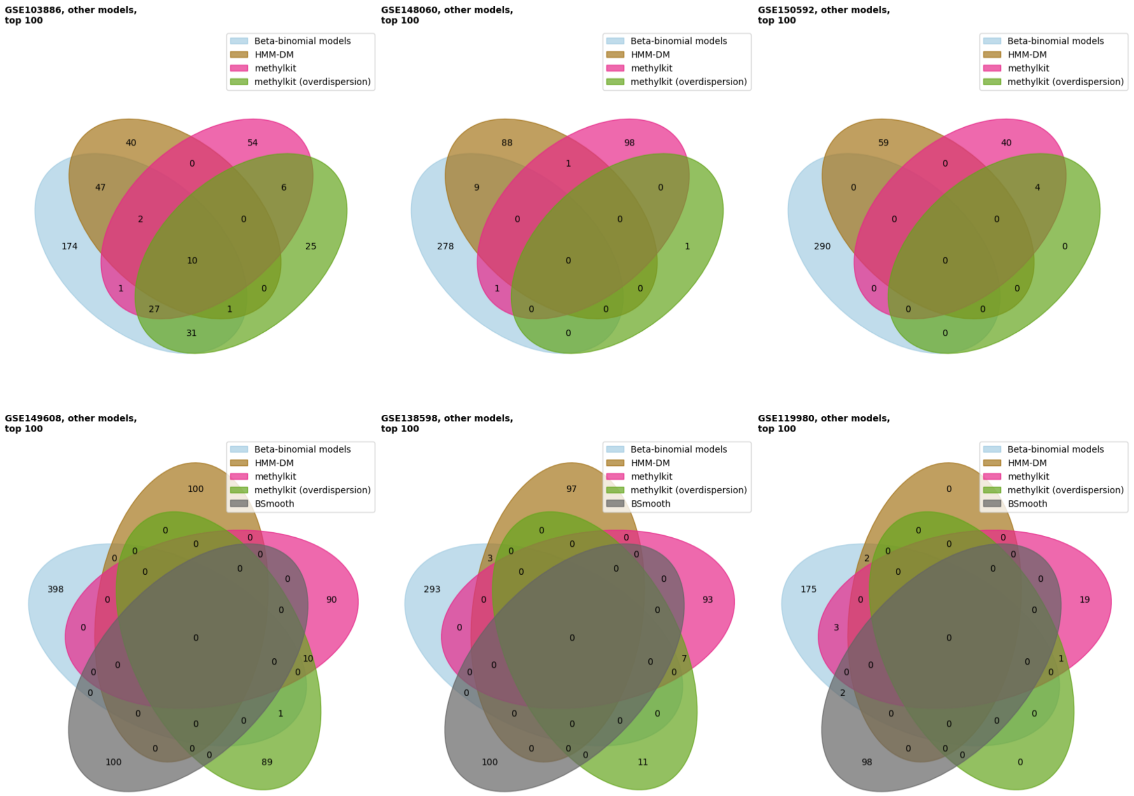

Supplementary Figure S9: Venn diagrams for the union of beta-binomial methods DMCs (blue) and other evaluated NGS methods (the top 100 DMC signatures).

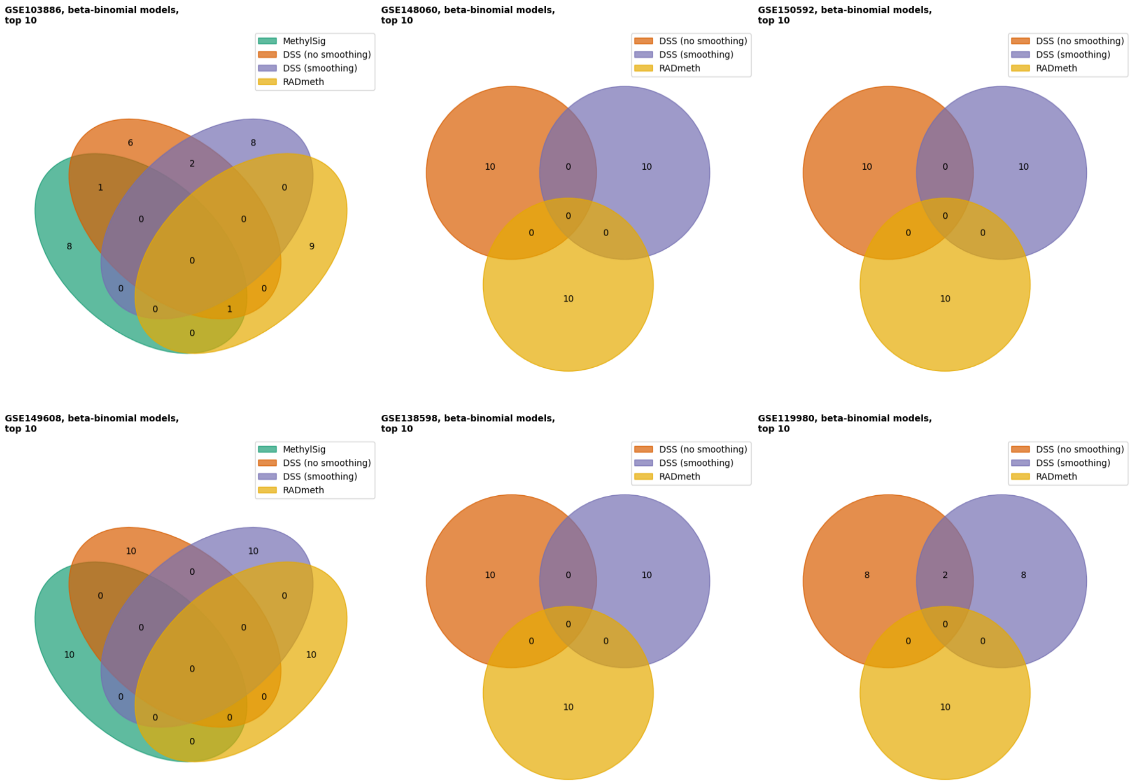

Supplementary Figure S10: Venn diagrams for NGS beta-binomial DM methods (the top 10 DMC signatures).

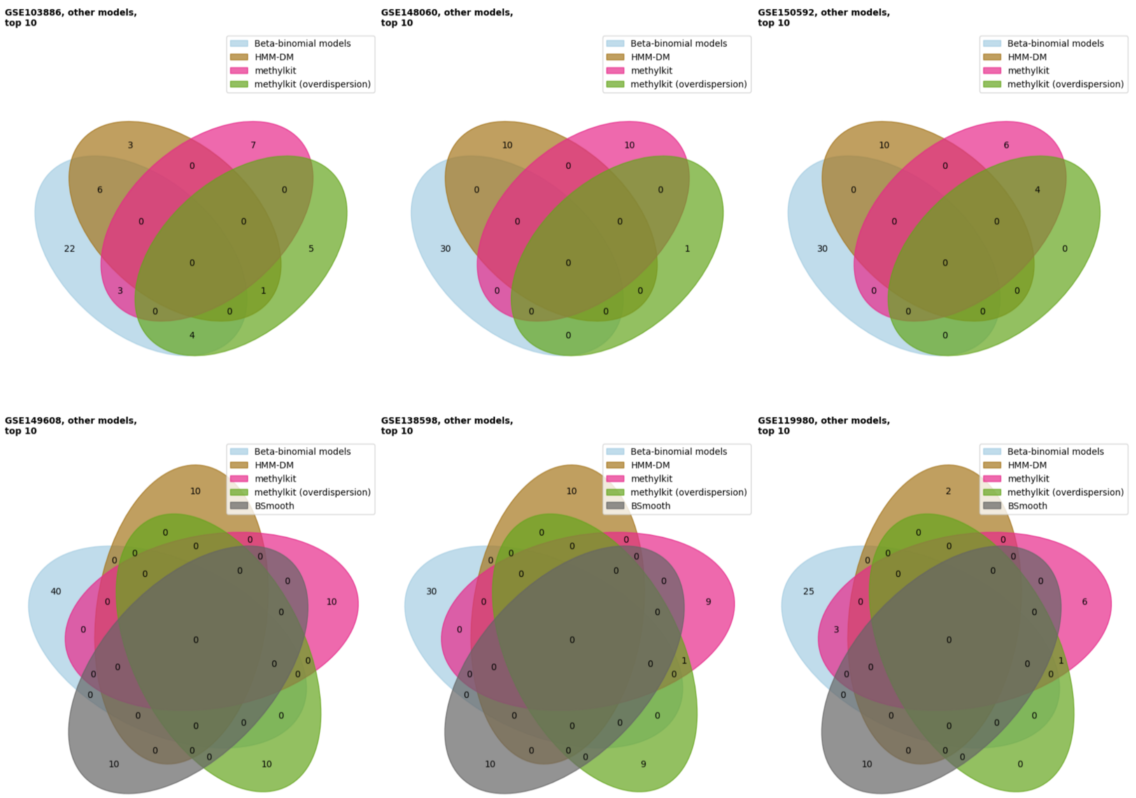

Supplementary Figure S11: Venn diagrams for the union of beta-binomial methods DMCs (blue) and other evaluated NGS methods (the top 10 DMC signatures).

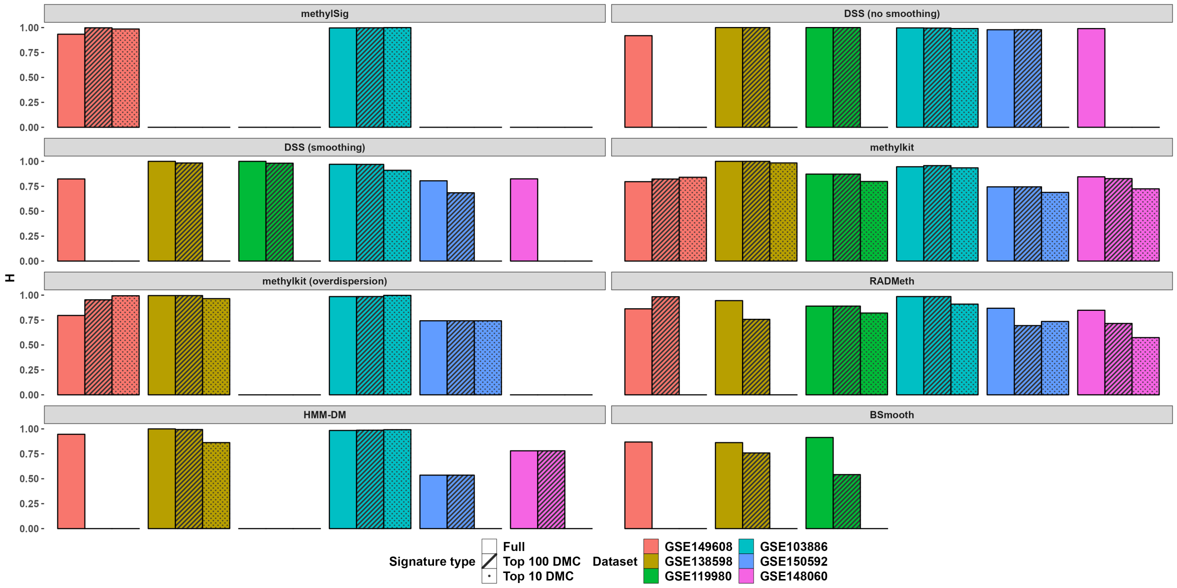

Supplementary Figure S12: Hobotnica metric values for each NGS method.

Supplementary Table S4: H-scores, lengths, and P-values for the full NGS signatures.

Dataset	Method	Length	H-score	P-value
GSE150592	methylSig	0	0	0
	DSS (no smoothing)	445	0.9785	0.0002
	DSS (smoothing)	27759	0.8049	0.0002
	methylkit	44	0.7443	0.0002
	methylkit (overdispersion)	4	0.7426	0.7426
	RADMeth	18267	0.8685	0.0002
	HMM-DM	59	0.5361	0.172
GSE149608	methylSig	5928190	0.9334	0.0002
	DSS (no smoothing)	6649145	0.9189	0.0002
	DSS (smoothing)	17844028	0.8236	0.0002
	methylkit	2050	0.7961	
	methylkit (overdispersion)	2050	0.7961	
	RADMeth	12257112	0.8626	0.0002
	HMM-DM	1922235	0.946	0.0002
	BSmooth	2082041	0.8683	0.0002
GSE148060	methylSig	0	0	
	DSS (no smoothing)	12448	0.9897	0.0002
	DSS (smoothing)	6860	0.8244	0.0002
	methylkit	511	0.8449	0.0002
	methylkit (overdispersion)	1	0	
	RADMeth	662	0.8483	0.0002
	HMM-DM	98	0.7803	0.0002
GSE138598	methylSig	0	0	0
	DSS (no smoothing)	4782	1	0.0002
	DSS (smoothing)	10214	1	0.0002
	methylkit	15227	1	0.0002
	methylkit (overdispersion)	18	0.9954	0.0002
	RADMeth	31727	0.9449	0.0002
	HMM-DM	518	1	0.0002
	BSmooth	40478	0.8624	0.0002
GSE119980	methylSig	0	0	
	DSS (no smoothing)	57	1	
	DSS (smoothing)	650	1	0.0002
	methylkit	23	0.8722	
	methylkit (overdispersion)	1	0	
	RADMeth	52	0.8898	
	HMM-DM	2	0	
	BSmooth	1151	0.9139	0.0002
GSE103886	methylSig	202	0.9965	0.0002
	DSS (no smoothing)	691	0.997	0.0002
	DSS (smoothing)	2945	0.9706	0.0002
	methylkit	1510	0.9459	0.0002
	methylkit (overdispersion)	561	0.9853	0.0002
	RADMeth	3670	0.9855	0.0002
	HMM-DM	155	0.9842	0.0002

Supplementary Table S5: H-scores, lengths, and P-values for the top 100 DMC NGS signatures.

Dataset	Method	Length	H-score	P-value
GSE150592	methylSig	0	0	0
	DSS (no smoothing)	100	0.9794	0.0002
	DSS (smoothing)	100	0.6847	0.0002
	methylkit	44	0.7443	0.0002
	methylkit (overdispersion)	4	0.7426	0.7426
	RADMeth	100	0.6944	0.0002
	HMM-DM	59	0.5361	0.1714
GSE149608	methylSig	100	0.9978	
	DSS (no smoothing)	100	0	
	DSS (smoothing)	100	0	
	methylkit	100	0.8229	
	methylkit (overdispersion)	100	0.9527	
	RADMeth	100	0.9837	
	HMM-DM	100	0	
	BSmooth	100	0	
GSE148060	methylSig	0	0	
	DSS (no smoothing)	100	0	
	DSS (smoothing)	100	0	
	methylkit	100	0.827	0.0002
	methylkit (overdispersion)	1	0	
	RADMeth	100	0.7156	0.0002
	HMM-DM	98	0.7803	0.0002
GSE138598	methylSig	0	0	0
	DSS (no smoothing)	100	1	0.0002
	DSS (smoothing)	100	0.9837	0.0002
	methylkit	100	1	0.0002
	methylkit (overdispersion)	18	0.9954	0.0002
	RADMeth	100	0.7572	0.0002
	HMM-DM	100	0.9937	0.0002
	BSmooth	100	0.7587	0.0002
GSE119980	methylSig	0	0	
	DSS (no smoothing)	57	1	
	DSS (smoothing)	100	0.9815	0.0002
	methylkit	23	0.8722	
	methylkit (overdispersion)	1	0	
	RADMeth	52	0.8898	
	HMM-DM	2	0	
	BSmooth	100	0.5417	0.2761
GSE103886	methylSig	100	0.9979	0.0002
	DSS (no smoothing)	100	0.9956	0.0002
	DSS (smoothing)	100	0.9696	0.0002
	methylkit	100	0.9571	0.0002
	methylkit (overdispersion)	100	0.9859	0.0002
	RADMeth	100	0.9856	0.0002
	HMM-DM	100	0.9872	0.0002

Supplementary Table S6: H-scores, lengths, and P-values for the top 10 DMC NGS signatures.

Dataset	Method	Length	H-score	P-value
GSE150592	methylSig	0	0	0
	DSS (no smoothing)	10	0	0.0002
	DSS (smoothing)	10	0	0.0002
	methylkit	10	0.6885	0.0002
	methylkit (overdispersion)	4	0.7426	0.7426
	RADMeth	10	0.7356	0.0002
	HMM-DM	10	0	0.1714
GSE149608	methylSig	10	0.9857	
	DSS (no smoothing)	10	0	
	DSS (smoothing)	10	0	
	methylkit	10	0.8404	
	methylkit (overdispersion)	10	0.9933	
	RADMeth	10	0	
	HMM-DM	10	0	
	BSmooth	10	0	
GSE148060	methylSig	0	0	
	DSS (no smoothing)	10	0	
	DSS (smoothing)	10	0	
	methylkit	10	0.7243	
	methylkit (overdispersion)	1	0	
	RADMeth	10	0.5739	
	HMM-DM	10	0	
GSE138598	methylSig	0	0	
	DSS (no smoothing)	10	0	
	DSS (smoothing)	10	0	
	methylkit	10	0.9842	
	methylkit (overdispersion)	10	0.9648	
	RADMeth	10	0	
	HMM-DM	10	0.8626	
	BSmooth	10	0	
GSE119980	methylSig	0	0	
	DSS (no smoothing)	10	0	
	DSS (smoothing)	10	0	
	methylkit	10	0.7981	
	methylkit (overdispersion)	1	0	
	RADMeth	10	0.8204	
	HMM-DM	2	0	
	BSmooth	10	0	
GSE103886	methylSig	10	1	
	DSS (no smoothing)	10	0.9903	
	DSS (smoothing)	10	0.9105	
	methylkit	10	0.9349	
	methylkit (overdispersion)	10	0.9978	
	RADMeth	10	0.9098	0.0002
	HMM-DM	10	0.9919	

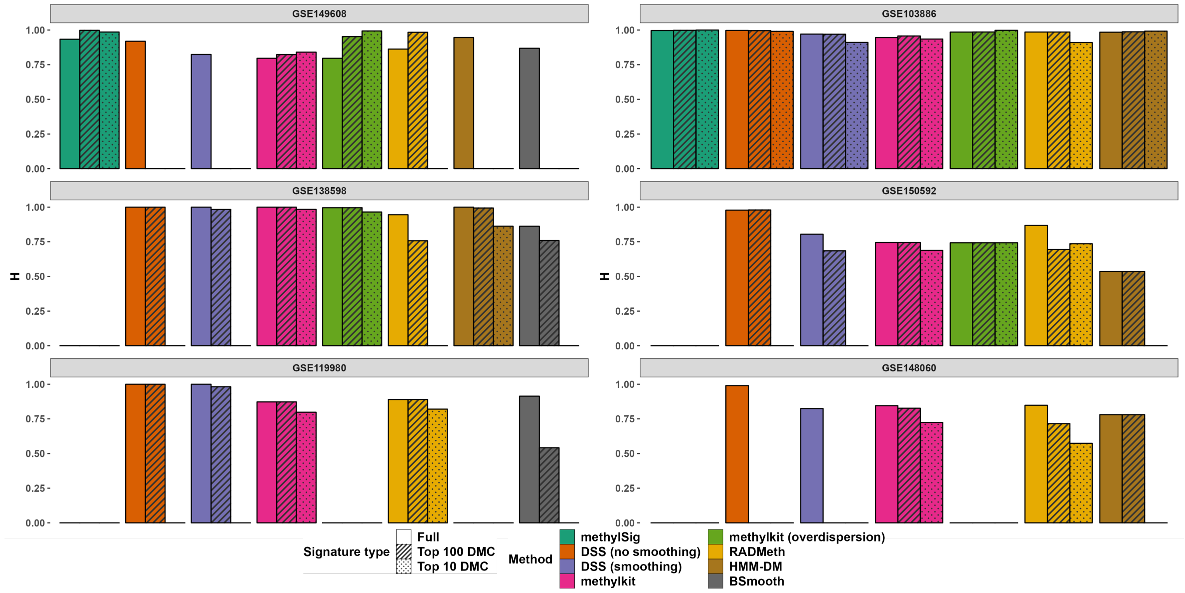

Supplementary Figure S13: Hobotnica metric values for each NGS dataset.

Supplementary Table S7: Results of Friedman test used to compare H-scores of signatures resulting from different methods.

Data type	Signature	Sample size	Shapiro-Wilk p-value	Friedman p-value
Microarray	full	16x4	2.186e-10	0.6325
Microarray	top 100 DMP	16x4	6.147e-12	0.1049
Microarray	top 10 DMP	16x4	5.56e-12	0.1527
WGBS	full	3x8	8.529e-07	0.4382
WGBS	top 100 DMC	3x8	1.789e-05	0.7149
WGBS	top 10 DMC	3x8	1.508e-06	0.171
RRBS	full	3x7	2.042e-05	0.09196
RRBS	top 100 DMC	3x7	0.0001883	0.8041
RRBS	top 10 DMC	3x7	0.0001782	0.572
WGBS and RRBS (without BSmooth)	full	6x7	1.227e-08	0.04019
WGBS and RRBS (without BSmooth)	top 100 DMC	6x7	1.462e-07	0.5936
WGBS and RRBS (without BSmooth)	top 10 DMC	6x7	1.247e-07	0.05854

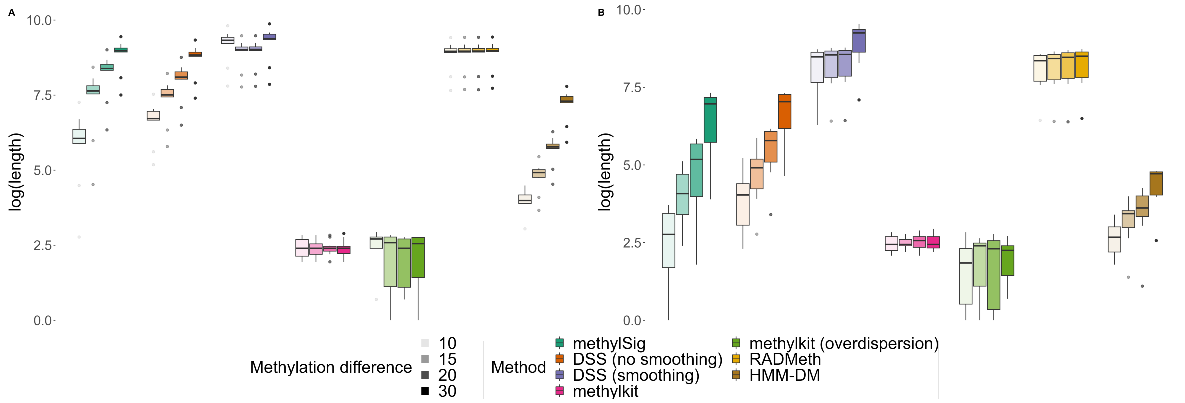

Supplementary Figure S14: Signature length for simulated data (**A**): The first group of datasets. (**B**): The second group of datasets.

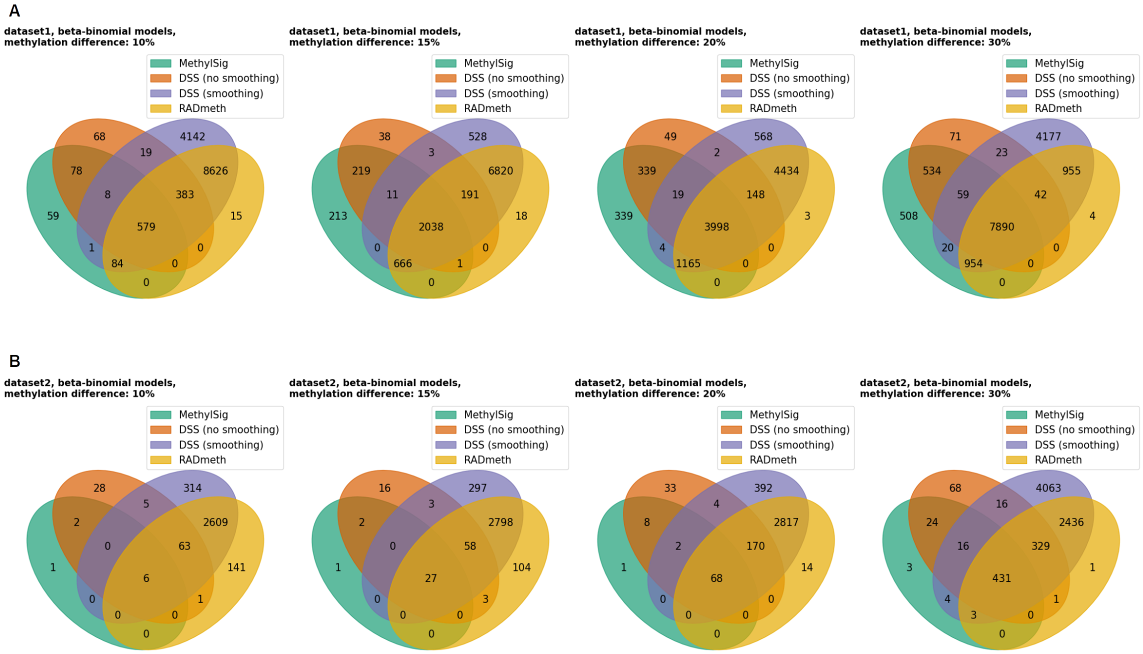

Supplementary Figure S15: Venn diagrams for beta-binomial DM methods (the full signatures, simulated data) (**A**): The first group of datasets. (**B**): The second group of datasets.

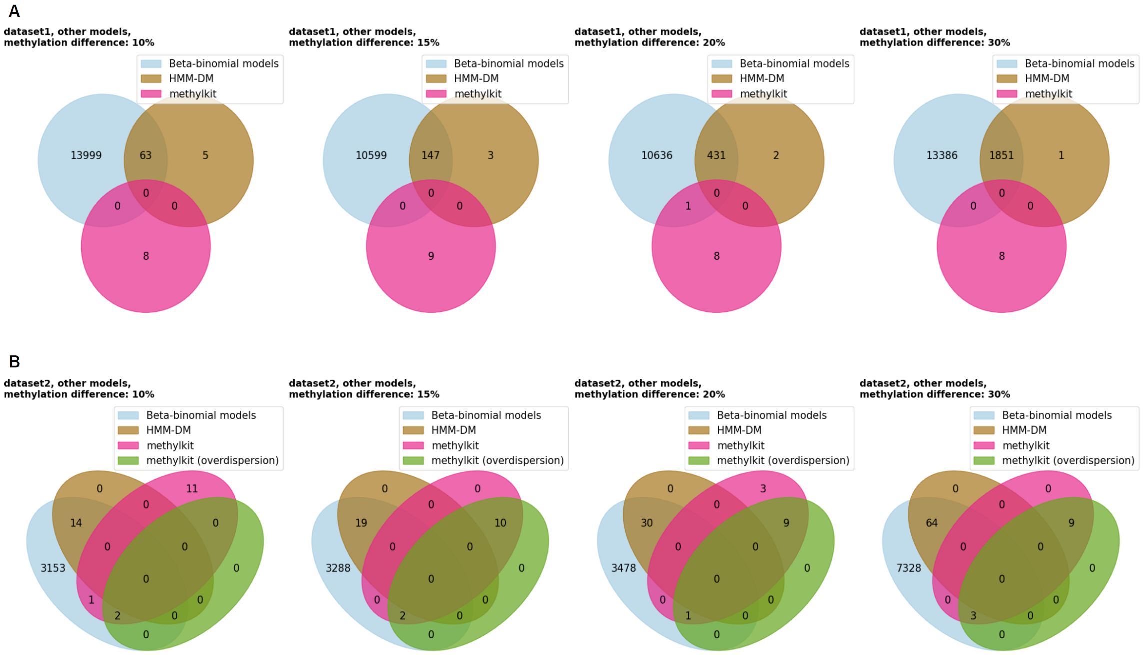

Supplementary Figure S16: Venn diagrams for the union of beta-binomial methods DMCs (blue) and other evaluated methods (the full signatures, simulated data) (**A**): The first group of datasets. (**B**): The second group of datasets.

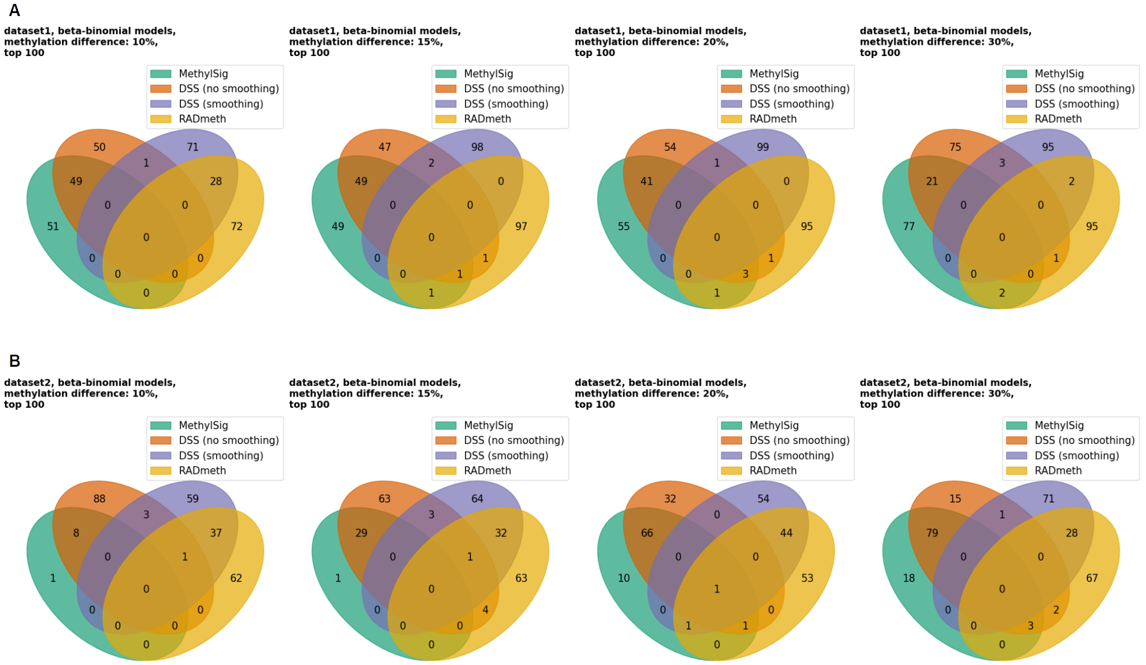

Supplementary Figure S17: Venn diagrams for beta-binomial DM methods (the top 100 DMC signatures, simulated data) (**A**): The first group of datasets. (**B**): The second group of datasets.

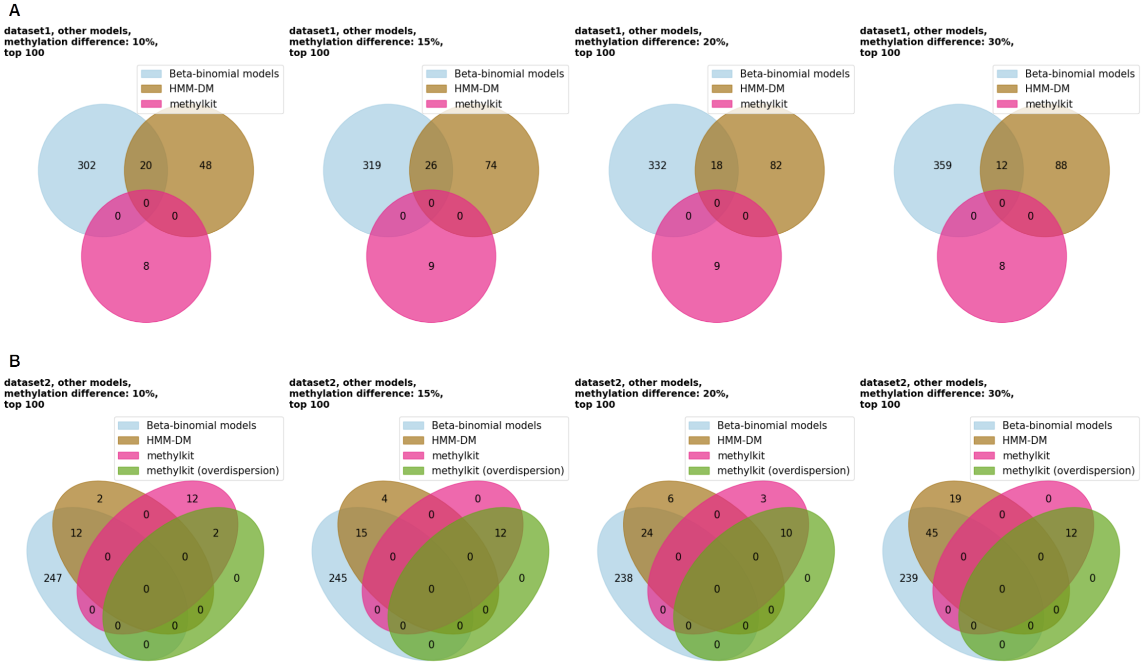

Supplementary Figure S18: Venn diagrams for the union of beta-binomial methods DMCs (blue) and other evaluated methods (the top 100 DMC signatures, simulated data) (**A**): The first group of datasets. (**B**): The second group of datasets.

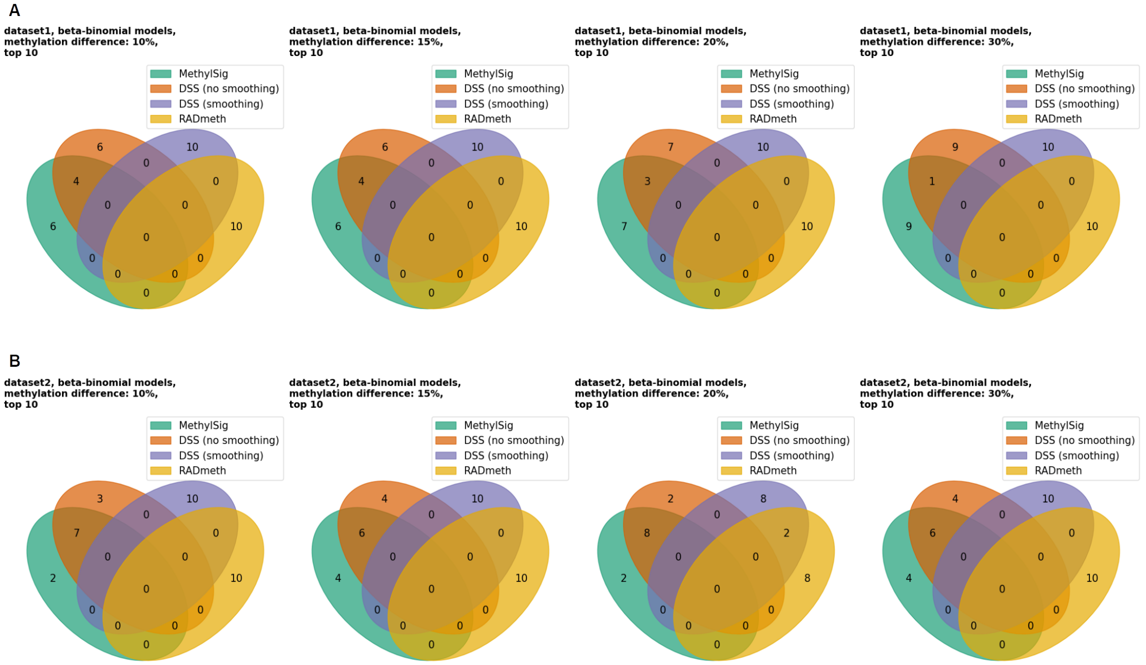

Supplementary Figure S19: Venn diagrams for beta-binomial DM methods (the top 10 DMC signatures, simulated data) (**A**): The first group of datasets. (**B**): The second group of datasets.

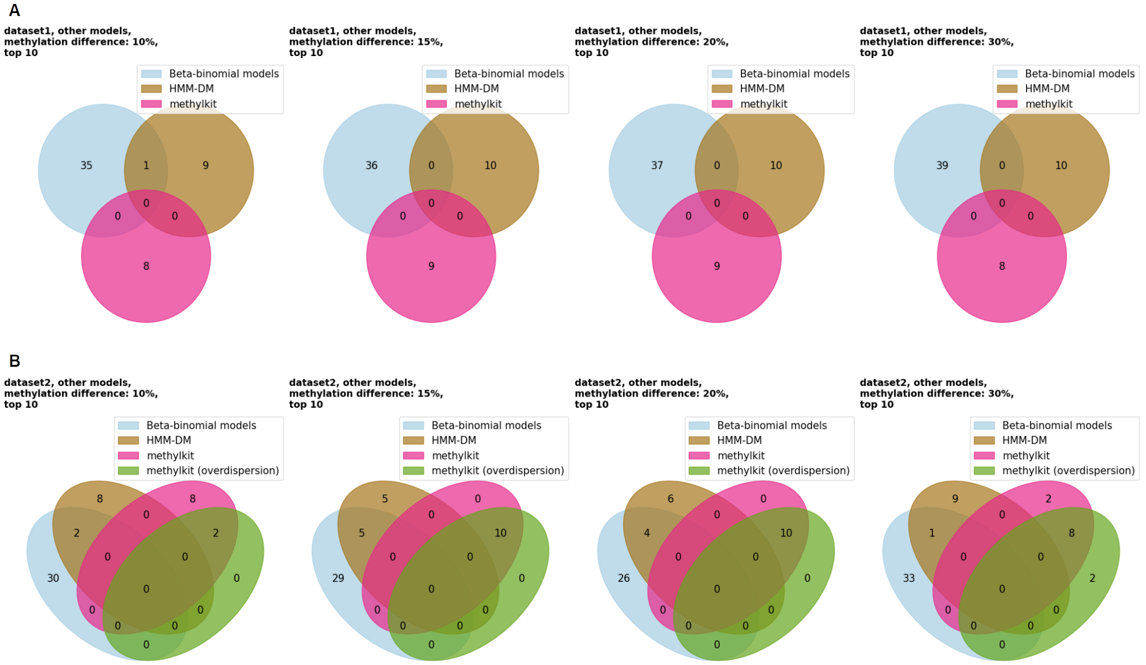

Supplementary Figure S20: Venn diagrams for the union of beta-binomial methods DMCs (blue) and other evaluated methods (the top 10 DMC signatures, simulated data) (**A**): The first group of datasets. (**B**): The second group of datasets.

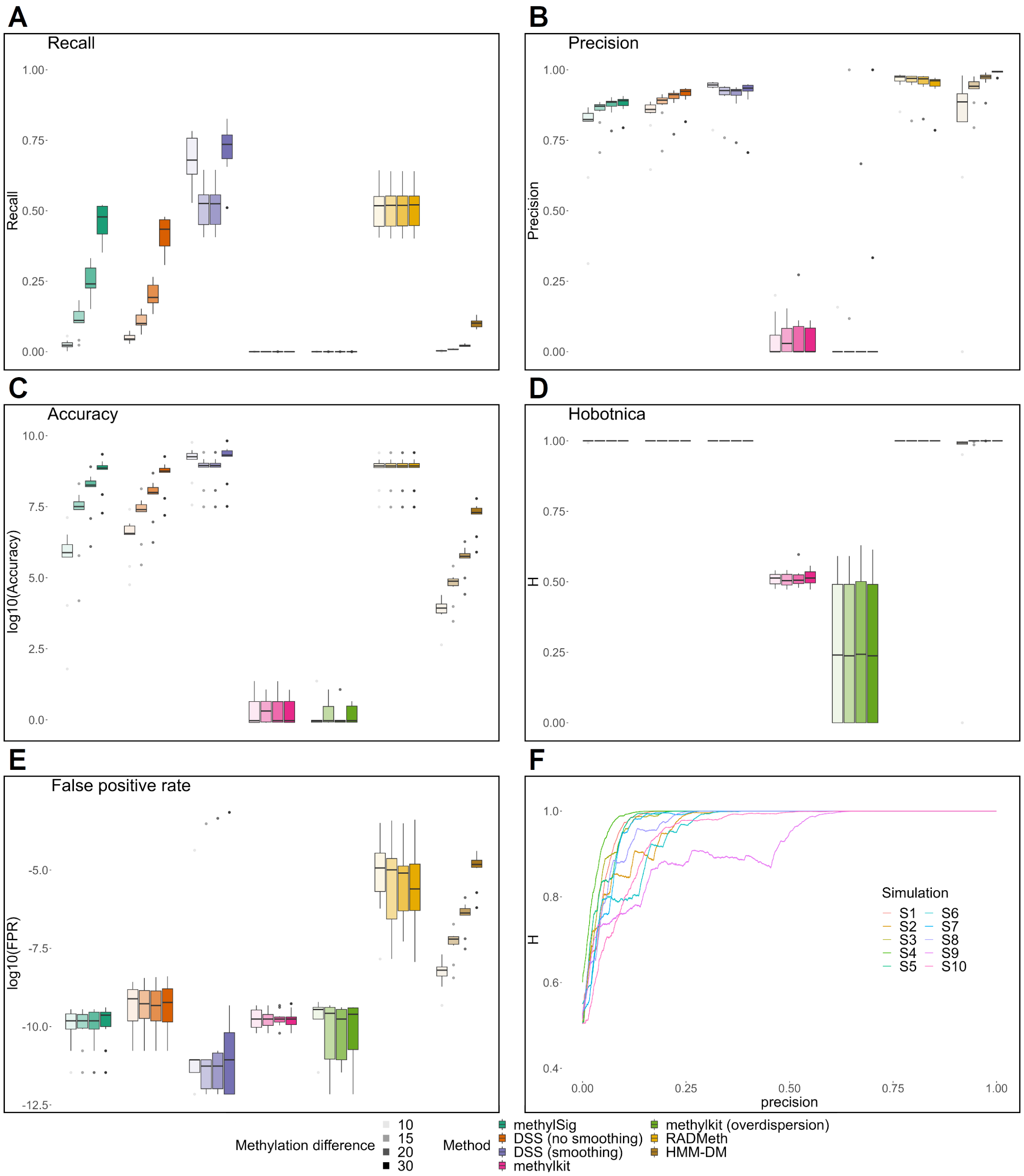

Supplementary Figure S21: Results for DM analysis on simulated data (the first group of datasets simulated with default parameters: (**A**): Recall, (**B**): Precision, (**C**): Accuracy for signatures produced from datasets simulated with methylation differences (**D**): Hobotnica for signatures produced from datasets simulated with methylation differences. (**E**): False positive rate for signatures obtained after permuting sample labels for each simulated dataset. (**F**): Dependence of the H-score on the proportion of true positive DMC included in the signature. A signature had the same length as a ground truth signature for each simulation with an average difference in methylation levels of 0.1.

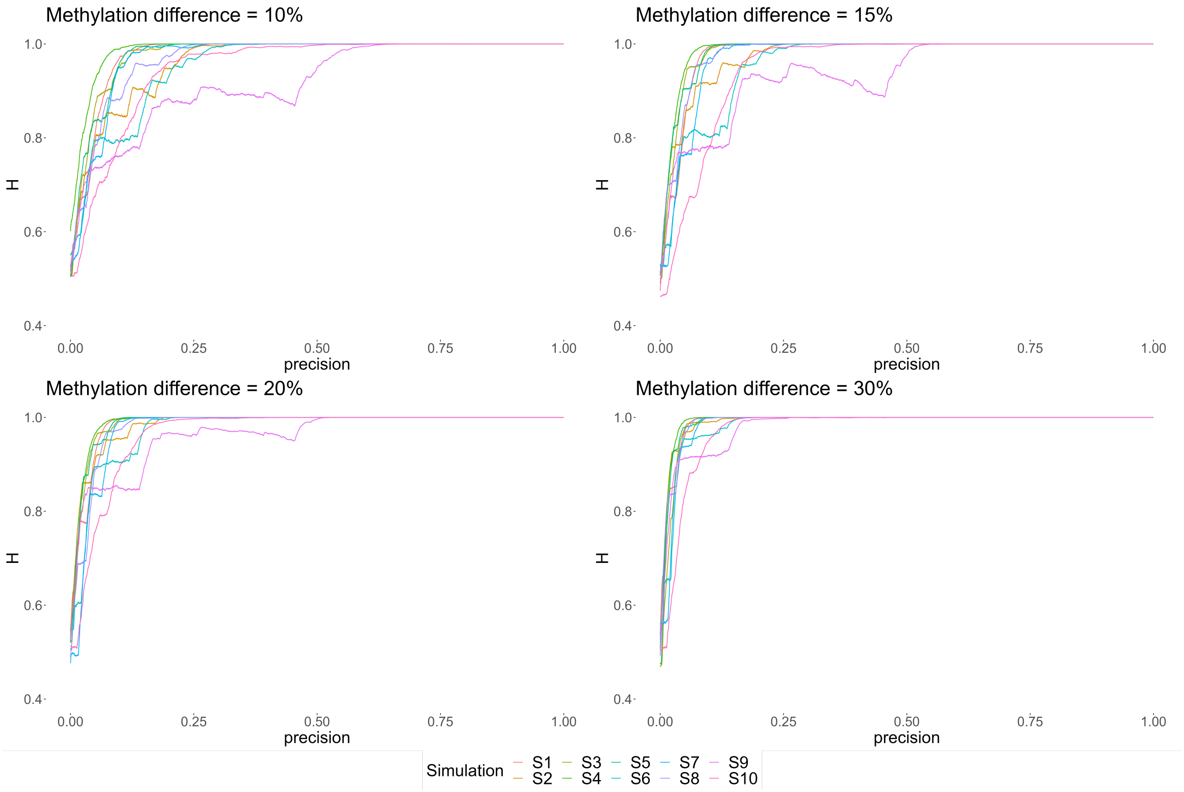

Supplementary Figure S22: Dependence of the H-score on the proportion of true positive DMC included in the signature, the first group of datasets. A signature had the same length as a ground truth signature for each simulation.

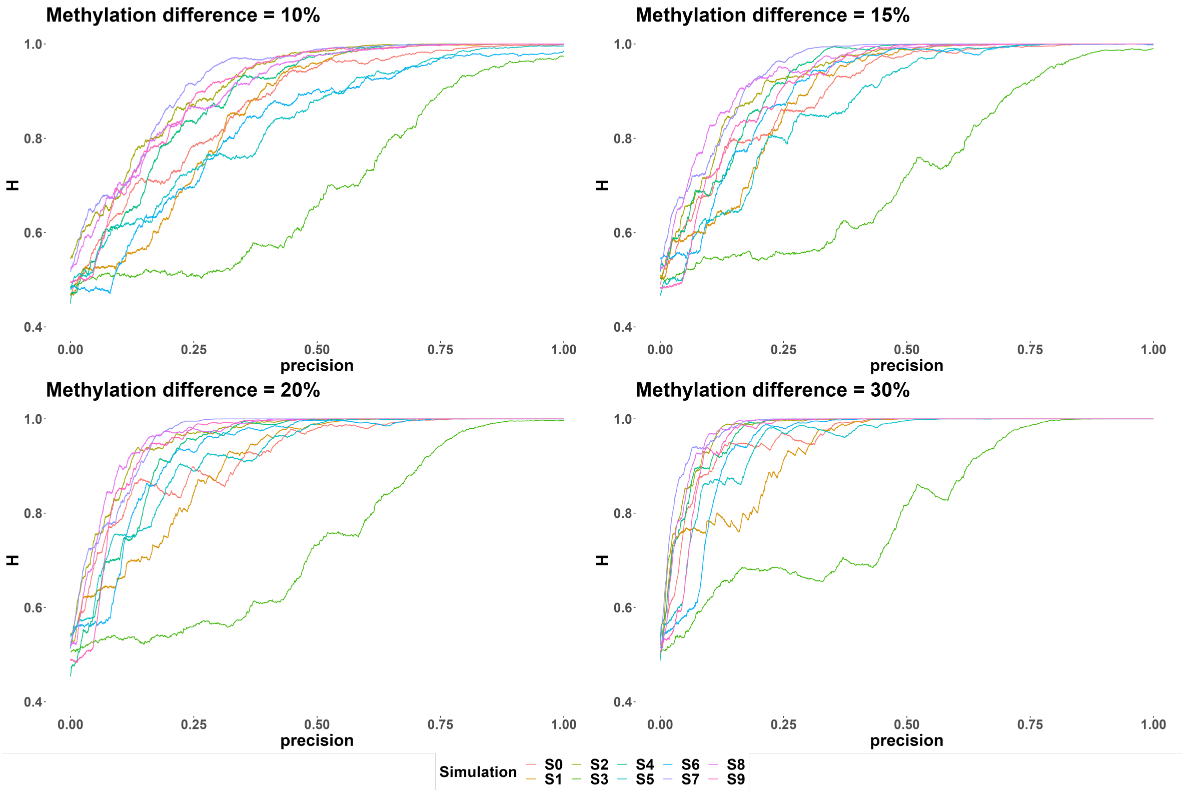

Supplementary Figure S23: Dependence of the H-score on the proportion of true positive DMC included in the signature, the second group of datasets. A signature had the same length as a ground truth signature for each simulation.

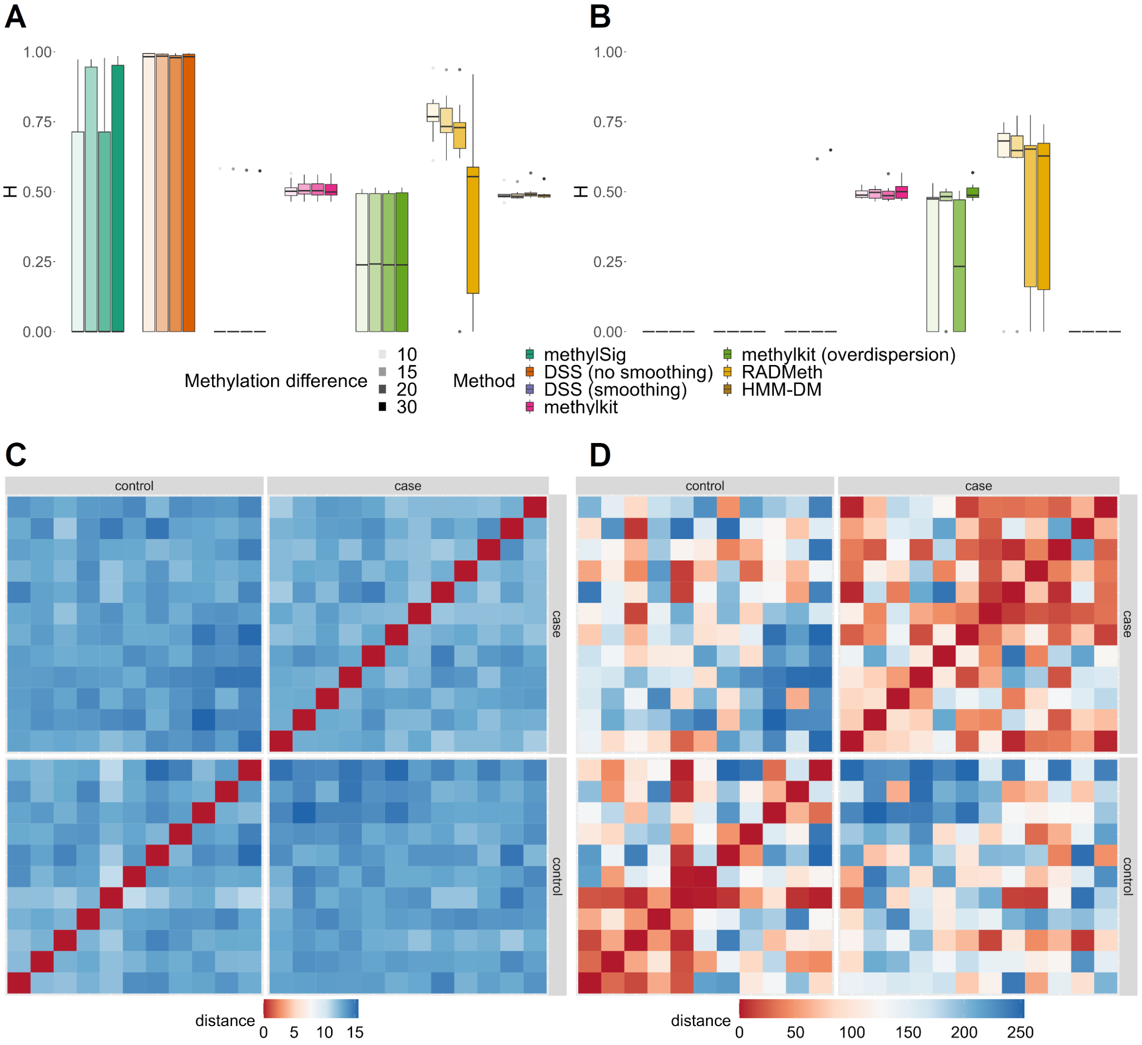

Supplementary Figure S24: Hobotnica calculation for label permutation (**A**): H-scores for label permutation signatures, the first group of datasets (**B**): H-scores for label permutation signatures, the second group of datasets (**C**): The distances between the vectors of methylation levels for RADmeth signature, the second group of datasets, simulation 1, methylation difference 0.15 (H-score = 0.7224) (**D**): The ranks of distances for RADmeth signature, the second group of datasets, simulation 1, methylation difference 0.15.

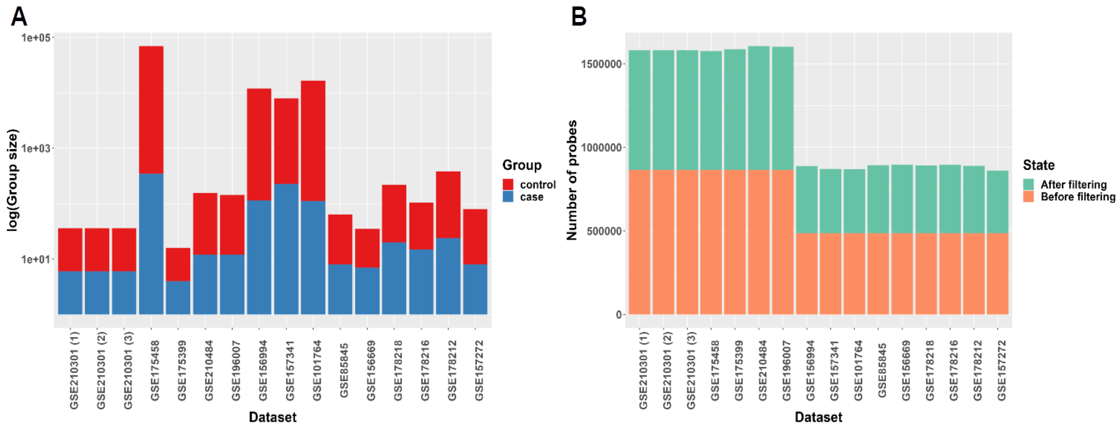

Supplementary Figure S25: Microarray data preprocessing (**A**): Dataset-wise per-group number of samples. (**B**): Number of methylation probes before and after filtering.

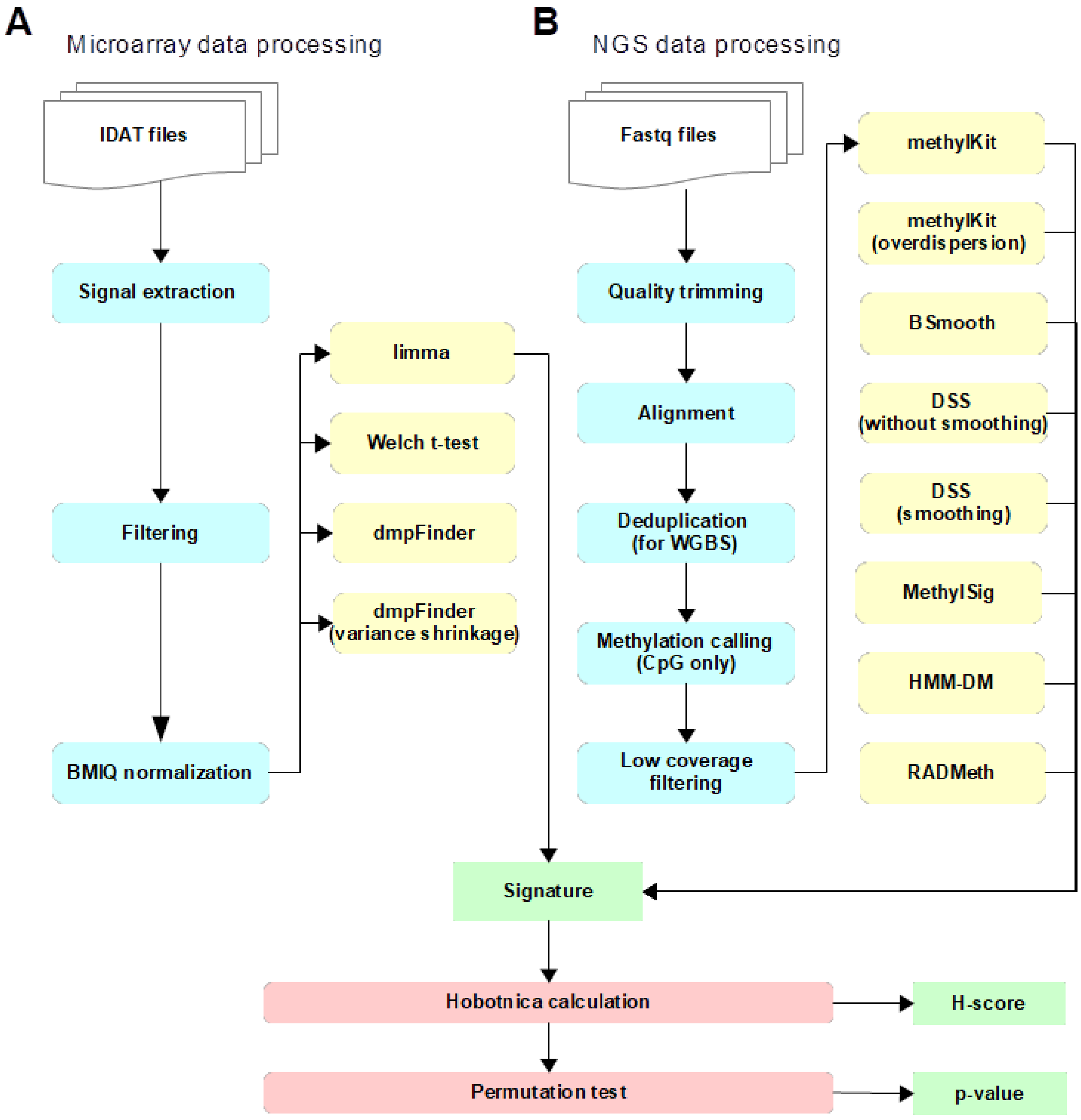

Supplementary Figure S26: Processing workflows for microarray and NGS data

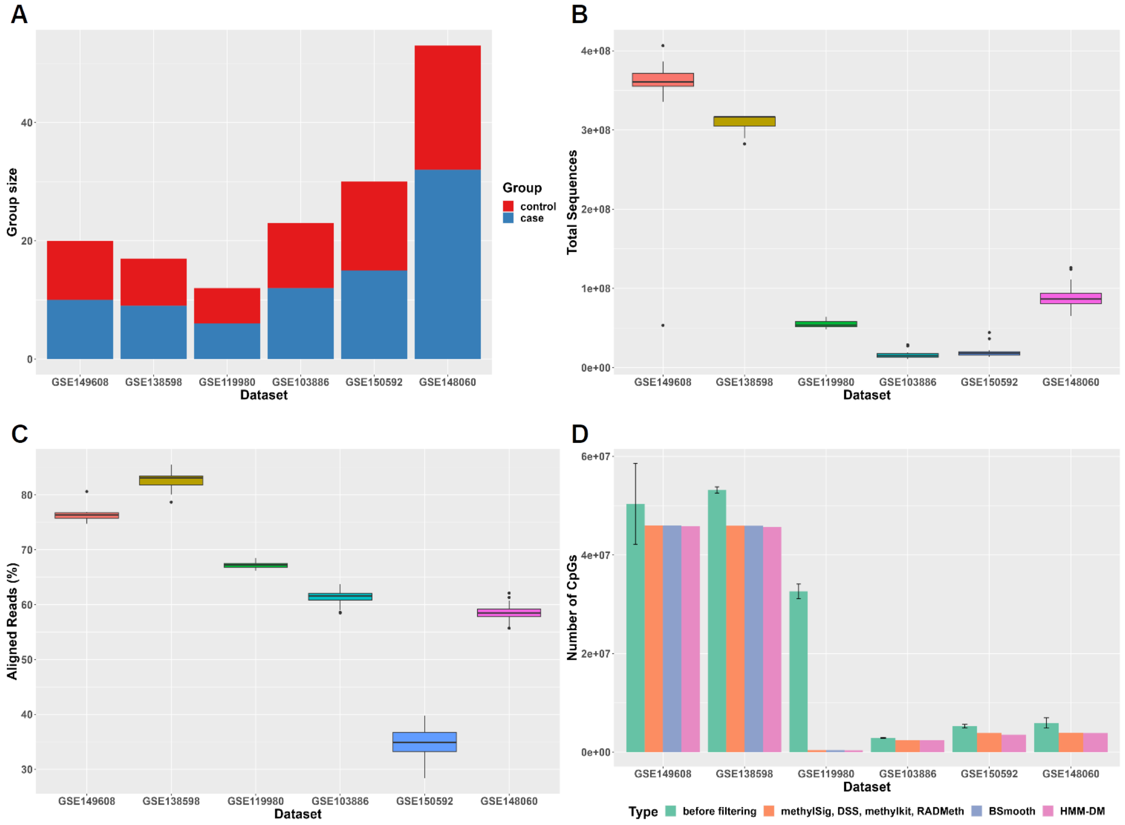

Supplementary Figure S27: NGS data preprocessing (**A**): Dataset-wise per-group number of samples. (**B**): Number of reads per FASTQ file. (**C**): Percent of uniquely aligned reads. (**D**): The number of CpG sites before filtering (green) and after filtering passed as an input to each DM method.
